# Mechanical-induced bone remodeling does not depend on *Piezo1* in dentoalveolar hard tissue

**DOI:** 10.1038/s41598-023-36699-9

**Published:** 2023-06-12

**Authors:** Cita Nottmeier, Josef Lavicky, Marcos Gonzalez Lopez, Sarah Knauth, Bärbel Kahl-Nieke, Michael Amling, Thorsten Schinke, Jill Helms, Jan Krivanek, Till Koehne, Julian Petersen

**Affiliations:** 1 Department of Orthodontics, University of Leipzig Medical Center, Saxony, Germany; 2 Department of Histology and Embryology, Faculty of Medicine, Masaryk University, Brno, Czech Republic; 3 Department of Orthodontics, University Medical Center Hamburg-Eppendorf, Hamburg, Germany; 4 Institute of Osteology and Biomechanics, University Medical Center Hamburg-Eppendorf, Hamburg, Germany; 5 Division of Plastic and Reconstructive Surgery, Department of Surgery, Stanford School of Medicine, Stanford University, Palo Alto, CA USA

**Keywords:** Bone remodelling, Bone, Bone quality and biomechanics, Ion channel signalling

## Abstract

Mechanosensory ion channels are proteins that are sensitive to mechanical forces. They are found in tissues throughout the body and play an important role in bone remodeling by sensing changes in mechanical stress and transmitting signals to bone-forming cells. Orthodontic tooth movement (OTM) is a prime example of mechanically induced bone remodeling. However, the cell-specific role of the ion channels *Piezo1* and *Piezo2* in OTM has not been investigated yet. Here we first identify the expression of PIEZO1/2 in the dentoalveolar hard tissues. Results showed that PIEZO1 was expressed in odontoblasts, osteoblasts, and osteocytes, while PIEZO2 was localized in odontoblasts and cementoblasts. We therefore used a *Piezo1*^*floxed/floxed*^ mouse model in combination with *Dmp1*^*cre*^ to inactivate *Piezo1* in mature osteoblasts/cementoblasts, osteocytes/cementocytes, and odontoblasts. Inactivation of *Piezo1* in these cells did not affect the overall morphology of the skull but caused significant bone loss in the craniofacial skeleton. Histological analysis revealed a significantly increased number of osteoclasts in *Piezo1*^*floxed/floxed*^*;Dmp1*^*cre*^ mice, while osteoblasts were not affected. Despite this increased number of osteoclasts, orthodontic tooth movement was not altered in these mice. Our results suggest that despite *Piezo1* being crucial for osteoclast function, it may be dispensable for mechanical sensing of bone remodeling.

## Introduction

Bone homeostasis is a complex process controlled by both intrinsic and extrinsic mechanisms^1^. A striking example of the importance of extrinsic mechanism can be seen in astronauts who develop progressive osteopenia under conditions of weightlessness^2^. These mechanical stimuli (or lack thereof) influence the differentiation and activity of bone-resorbing osteoclasts and bone-forming osteoblasts^3,4^. In the past, it has been shown that osteocytes, as terminally differentiated osteoblasts embedded in the bone matrix, are crucial for the perception of mechanical stimuli. The osteocytes can sense the mechanical stimuli and generate signals that induce osteoblast formation^5–7^.

Osteocytes also appear to play a crucial role in bone remodeling processes during orthodontic tooth movement (OTM), which is an illustrative example of mechanically induced bone remodeling^8^. Here, extrinsic forces act on the tooth, forming pressure and tension zones in the cells of the periodontal ligament (PDL) and the surrounding bone. In the pressure zones, the compression of the periodontium results in inflammatory-mediated osteoclast formation and consequent resorption of the adjacent alveolar bone. In the tension zone, the mechanical stimulus results in increased osteoblast formation, which builds up the adjacent bone.

It was stated that the remodeling of the alveolar bone during orthodontic tooth movement is highly dependent on the RANKL expression of osteocytes^9^. Additionally, recent studies have shown that orthodontic tooth movement leads to apoptosis of osteocytes as a first step and subsequent activation of osteoclasts^10^. Another in vivo study showed how the ablation of osteocytes led to a significant decrease in orthodontic tooth movement in mice^11^. However, to what extent osteocytes sense the mechanical stimuli during orthodontic tooth movement and if this stimulus is necessary for bone remodeling is still not fully understood.

The group of mechanically activated ion channels, named Piezo channels, was first discovered in mammals by Coste et al.^12^. Previous studies have shown that these mechanosensitive ion channels *Piezo1* and *Piezo2* are expressed in different bone cells, such as osteoblasts, osteocytes, and osteoclasts, and are crucial for sensing mechanical stimuli and regulating the differentiation and activity of these cells^13–18^. Despite this research, the exact mechanisms by which *Piezo1* and *Piezo2* regulate bone remodeling are still not entirely known.

For example, in mouse models, cell-specific deletion of *Piezo1* in osteoblasts and osteocytes led to decreased bone density and increased bone porosity^16,17^. In addition to reduced bone formation, these mice also showed increased osteoclast differentiation factor RANKL and increased bone resorption. On the other hand, *Piezo2* seems to play a minor role in skeletal remodeling since mice with a cell-specific deficiency of *Piezo2* in osteoblast progenitor cells show only a negligible skeletal phenotype^17^.

Recently, the extent to which the expression of *Piezo1* and *Piezo2* in specific cell types regulates orthodontic tooth movement and, thus, bone remodeling has been questioned^19^. Indeed, a study showed that inhibition of *Piezo1* using the mechanosensitive ion channel inhibitor GsMTx4 reduced osteoclastogenesis and subsequently tooth movement in rats. However, due to the systemic application, no specific cell type that was affected by the inhibitor could be pointed out^20^. Furthermore, GsMTX4 is not specific for PIEZO proteins. It also inhibits other mechanosensitive ion channel such as transient receptor potential canonical (TRPC1) and TRPC6^21^. Therefore, in this study, we were particularly interested in the effect of a cell-specific deletion of *Piezo1* in osteocytes, which are essential for mechanically induced bone remodeling, using a tooth movement model in mice.

## Results

### Mechanosensing Ion Channels PIEZO1 and PIEZO2 are expressed in different dentoalveolar hard tissues

**Figure 1:**
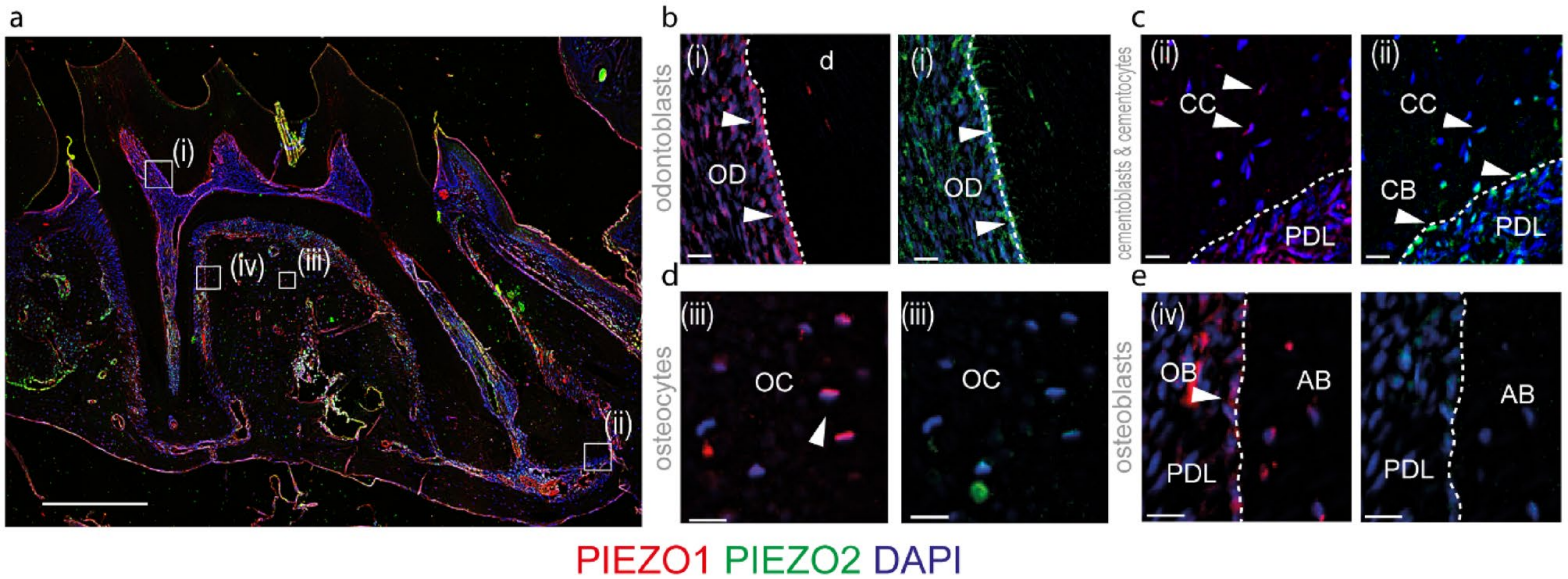
The expression of mechanosensitive ion channels PIEZO1 and PIEZO2 in dentoalveolar hard tissues. (**a**) Overview of an antibody staining for PIEZO1 and PIEZO 2 on a paraffine section of a control mouse. Scale bar = 500 µm. Zoom ins (**b**–**e**) show the expression of PIEZO1 and PIEZO2 in cells of the dentoalveolar tissues like odontoblasts (OD) and cementoblasts (CB), and cementocytes (CC) (**b**,**c**). Osteocytes (OC) and Osteoblasts (OB) show a higher expression of PIEZO1, whereas the expression of PIEZO2 is not evident (**d**,**e**). Scale bars = 20 µm.

To investigate how the mechanosensitive ion channels *Piezo1* and *Piezo2* can impact dentoalveolar hard tissues during orthodontic tooth movement, we first explored the specific expression pattern of PIEZO1 and PIEZO2 using antibody staining on paraffin sections of control animals (Fig. 1a). Here the staining shows the expression of PIEZO1 in cells like odontoblasts, cementocytes, osteocytes, and osteoblasts, whereas the expression of PIEZO2 was mainly observed in odontoblasts and cementoblasts (Fig. 1b–e). Furthermore, knockout of *Piezo2* in *Dmp1* expressing cells does not lead to a significant effect on general skull morphology and bone volume^17^ shown in Supplementary Fig. 1.

**Figure 2:**
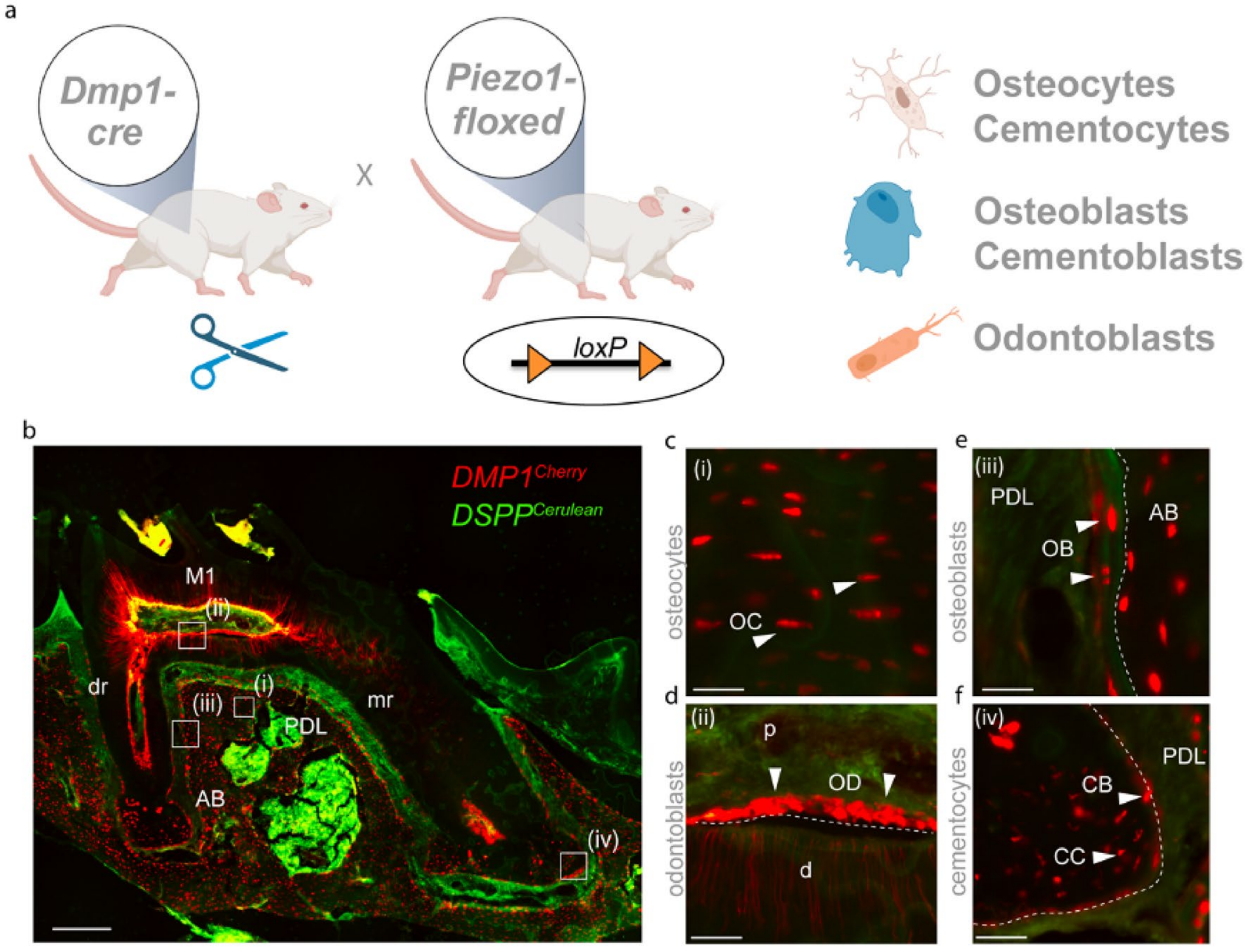
Dmp1 expressing cells can be found in dental hard tissues. (**a**) *Piezo1*^*floxed/floxed*^ mice and *Dmp1*^*cre*^ mice were mated using the *loxP* system to generate a tissue specific lack of *Piezo1* in *Dmp1* expressing cells like osteocytes, osteoblasts, odontoblasts, cementoblasts and cementocytes (Created with BioRender.com) (**b**) shows a sagittal section through the M1 of a *DSPP*^*Cerulean*^*;Dmp1*^*Cherry*^ Reporter Mouse using fluorescence imaging. scale bar = 250 µm. M1 = first Molar, *dr* distal root, *mr* mesial root, *AB* alveolar bone, *PDL* periodontal ligament. Details **(c-f)** show the expression of *Dmp1* in osteocytes (OC), osteoblasts (OB), odontoblasts (OD), cementoblasts (CB), and cementocytes (CC). *d* dentin, *p* pulp, *AB* alveolar bone, *PDL* periodontal ligament. scale bars = 50 µm.

We, therefore, focused our further investigations on *Piezo1* and used a *Cre/lox Piezo1* mouse model and the *Dmp1* promoter-driven expression of *Cre* recombinase to inactivate *Piezo1* expression in mature osteoblasts and osteocytes (Fig. 2a). We confirmed this specific expression of *Dmp1* using a *Dmp1*^*Cherry*^*;Dspp*^*Cerulean*^ mouse line showing the expression of *Dmp1*^Cherry^ transgenes in these particular dentoalveolar cell types. The transgenes were highly expressed not only in osteocytes but also osteoblasts within the alveolar bone, odontoblasts and cementocytes (Fig. 2b–f).

### Deletion of *Piezo1* in *Dmp1* expressing cells affects the alveolar bone volume

**Figure 3:**
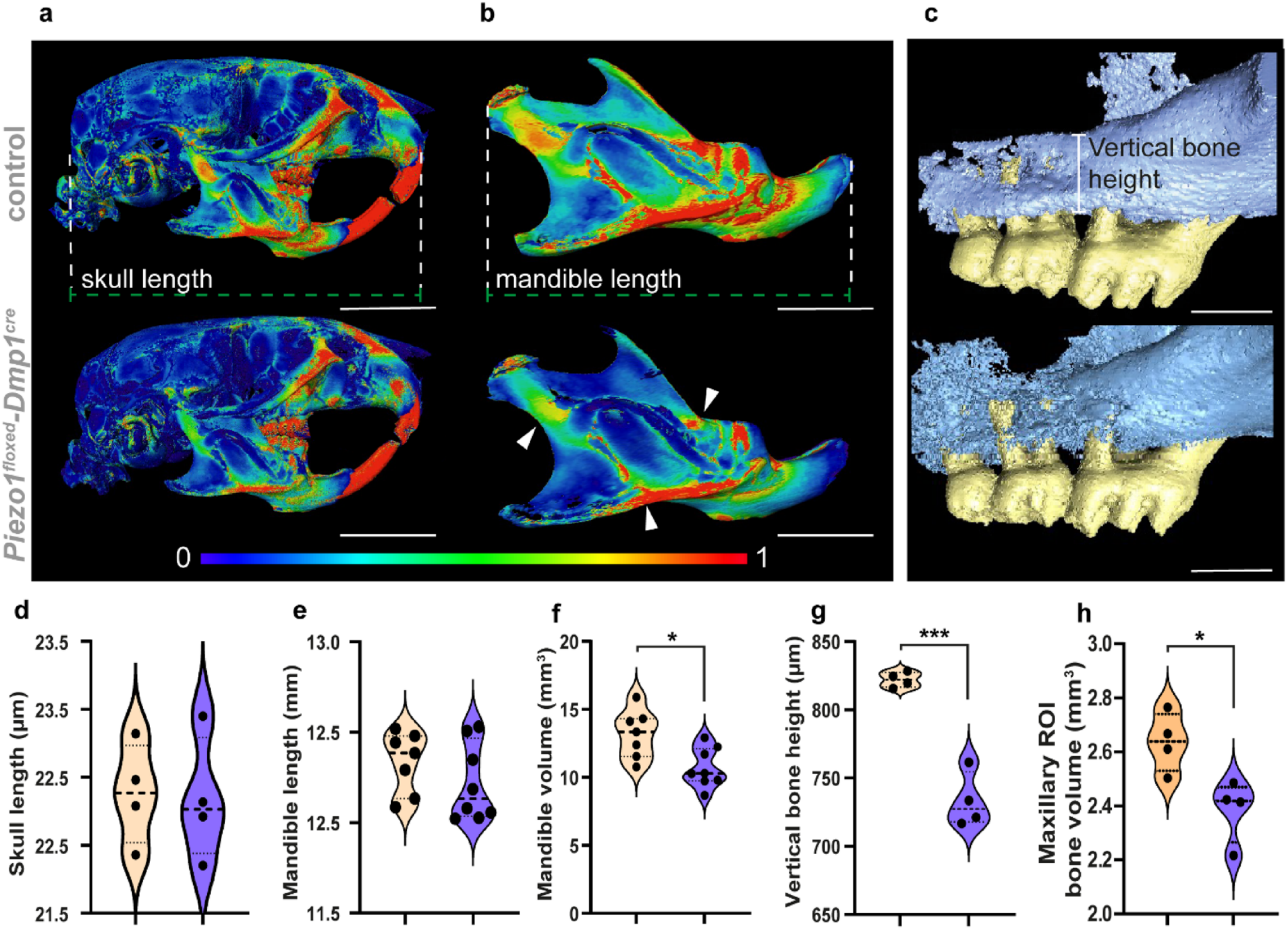
Deletion of *Piezo1* in *Dmp1* expressing cells leads to decreased alveolar bone volume in mice. (**a**,**b**) 3-dimensional reconstruction of Micro-Computed Tomography scans of Control (upper panels) and *Piezo1*^*floxed/floxed*^*;Dmp1*^*cre*^ (lower panels) mice. The Wall Thickness analysis of the skulls and mandibles shows, based on relative values, that the Bone thickness is decreased in *Piezo1*^*floxed/floxed*^*;Dmp1*^*cre*^ animals. This effect is also visible in the quantification of the Mandible Bone Volume (**f**) (n = 7). The skull and the mandible length do not differ between Control and *Piezo1*^*floxed/floxed*^*;Dmp1*^*cre*^ animals (**d**,**e**) (n = 4). (**c**) shows the bone loss in the maxillary bone. The vertical bone height (**g**) and also the bone volume in the maxilla (**h**) are significantly decreased compared to the values of control littermates (n = 4). Values are means ± SD. (*P < 0.05, ***P < 0.005*).* (Scale bars a = 5 mm, b = 2,5 mm, c = 1 mm).

Since mechanosensation is an important factor in bone homeostasis, we assumed that a loss of *Piezo1* would lead to a change in the skeletal morphology of the skull. To analyze this, we performed micro-computed tomography. In the 3-dimensional reconstructions of these scans, we observed that the inactivation of *Piezo1* in osteoblasts and osteocytes did not affect the morphology of the skull and the jaws (Fig. 3). Measurements of the skull length and the length of the mandible did not show differences between Piezo1 and control littermates (Fig. 3a,b,d,e). However, we observed significant bone loss in the craniofacial skeleton of *Piezo1*^*floxed/floxed*^*;Dmp1*^*cre*^ mice. The bone volume of the mandible and maxilla of *Piezo1*^*floxed/floxed*^*;Dmp1*^*cre*^ animals was significantly reduced compared to control littermates (Fig. 3a,b,f,h). In addition, we observed a loss of the vertical bone height between the tooth-bearing alveolar bones, resulting in a smaller root area covered by bone (Fig. 3c,g).

**Figure 4:**
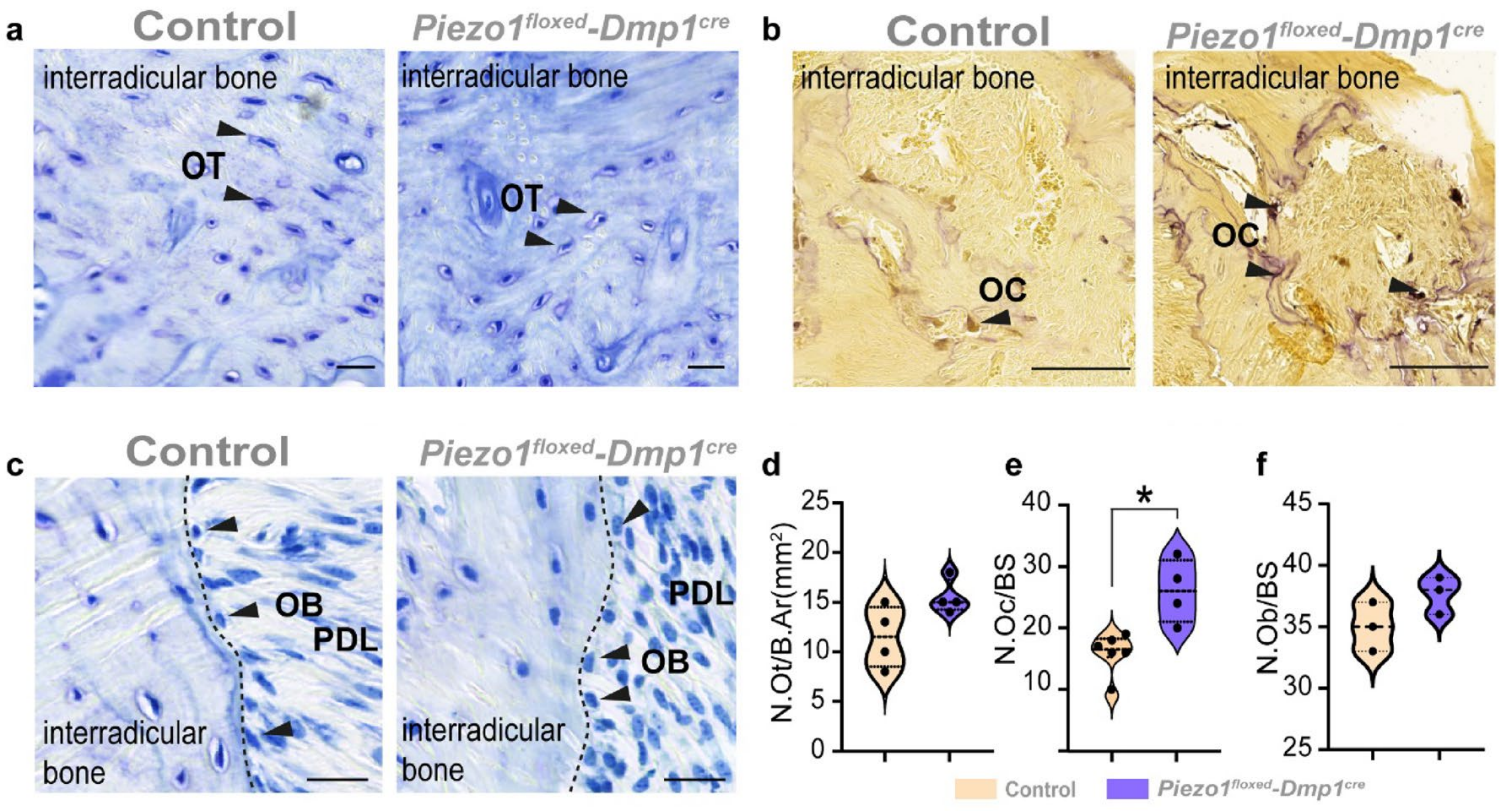
Deletion of *Piezo1* in *Dmp1* expressing cells leads to an increased osteoclast activity in the interradicular bone. (**a**) shows with the help of a toluidine-blue staining the osteocytes (OT) of the maxillary interradicular bone. Scale bars = 20 µm. They do not differ in number, nor in morphology, comparing the sections of control and *Piezo1*^*floxed/floxed*^*;Dmp1*^*cre*^ animals. N.Ot/B.Ar = Number of Osteocytes per Bone Area (**d**). The visualization of osteoclasts (OC) in the interradicular bone of control (left panel) and *Piezo1*^*floxed/floxed*^*;Dmp1*^*cre*^ animals (right panel) (**b**) was performed with a TRAP-staining. Scale bars = 50 µm. The black arrows show the osteoclasts, lining the bone surface. Quantification of them shows a basal increased osteoclast activity in *Piezo1*^*floxed/floxed*^*;Dmp1*^*cre*^ animals. N.Oc/BS = Number of Osteoclasts per Bone Surface (**e**). (**c**) shows the osteoblasts that are lining the bone surface of the interradicular bone. The number of osteoblasts in *Piezo1*^*floxed/floxed*^*;Dmp1*^*cre*^ animals is not affected compared to their control littermates. N.Ob/BS = Number of Osteoblasts per Bone Surface (**f**). Scale bars = 20 µm. Values are means ± SD. (*P < 0.05).

To further investigate these bone changes, we next performed histological analysis of the alveolar bone (Fig. 4). Morphometric analysis of osteocyte numbers on toluidine-blue stained sections showed no significant changes between *Piezo1*^*floxed/floxed*^*;Dmp1*^*cre*^ mice and control littermates. In contrast, TRAP staining revealed a significantly increased number of osteoclasts in *Piezo1*^*floxed/floxed*^*;Dmp1*^*cre*^ mice (Fig. 4a,b,d,e). The number of osteoblasts per bone surface, on the other hand, was not affected (Fig. 4c,f).

### The bone phenotype in *Piezo1*^*floxed/floxed*^*;Dmp1*^*cre*^ mice does not lead to altered tooth movement

**Figure 5:**
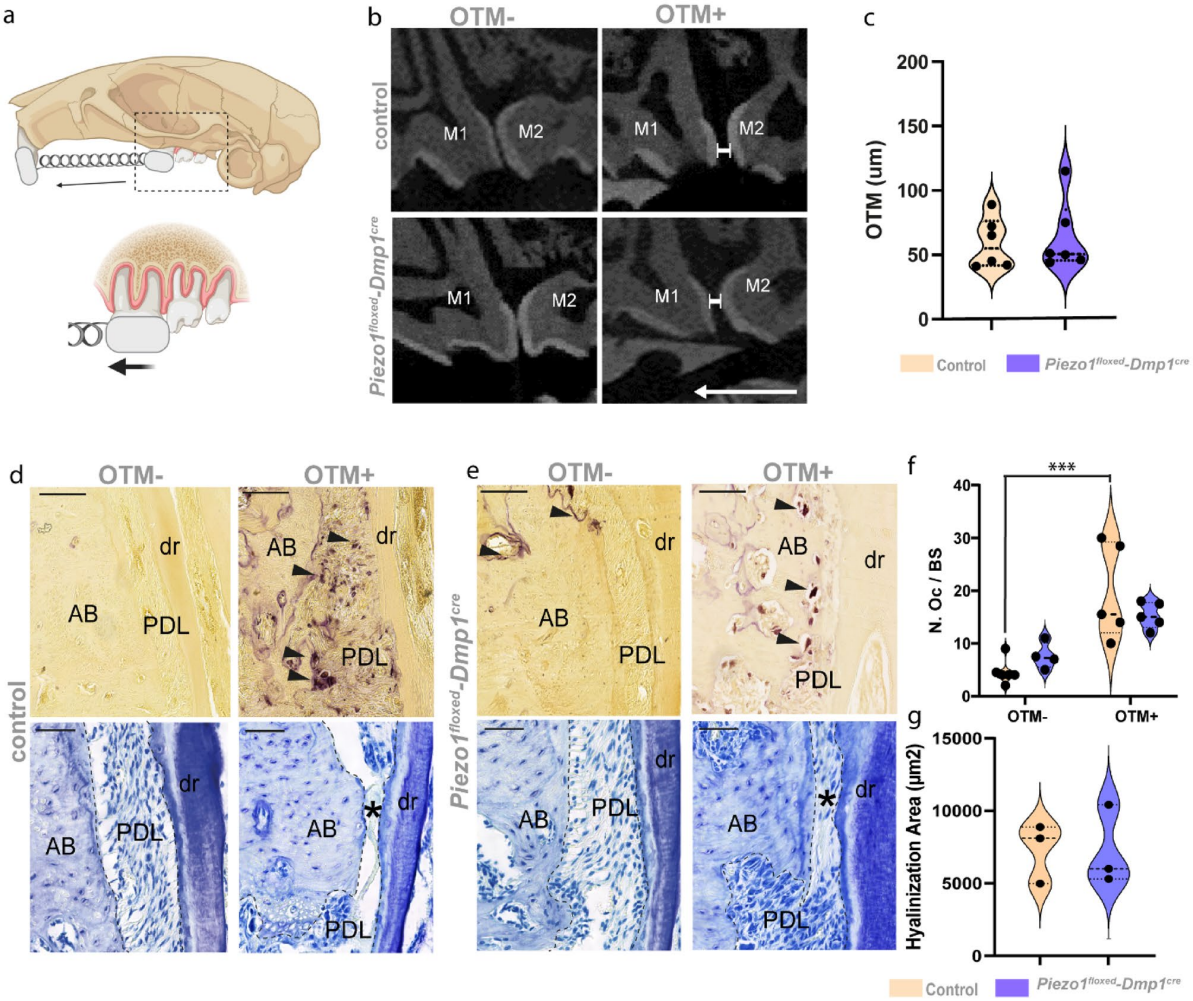
The amount of tooth movement in *Piezo1*^*floxed/floxed*^*;Dmp1*^*cre*^ mice is not affected. (**a**) Illustration of the OTM model in mice created with BioRender.com. A NiTi tension spring was attached with dental composite for 12 days between the M1 and the incisors with a force of 0.3N. The black arrow indicates the mesial direction of the force. The mice were sacrified after 12d of tooth movement. (**b**) µ-CT cross sections through M1-M3 showing the approximal gap between M1 and M2 after orthodontic tooth movement. (**c**) Quantification of the intercoronal distance between M1 and M2 after 12d of OTM showing no significant difference in control and *Piezo1*^*floxed/floxed*^*;Dmp1*^*cre*^ animals. (n = 6) *P < 0.05. (**d**) TRAP (upper panels) and toluidine blue staining (lower panels), showing the osteoclasts and the PDL fibroblasts in the pressure zone of the distal root of the M1. In the OTM- group, there is no osteoclast activity visible and the fibroblasts show a straight arrangement, as well as an intact insertion in the alveolar bone (AB). In the OTM + group, the pressure leads to a significant increase in osteoclast activity. *dr* distal root (**f**). Furthermore, it leads to a compression of the PDL fibroblasts with subsequent hyalinization of the PDL (black asterisk). (**e**) is showing the TRAP staining and toluidine blue staining for sections of *Piezo1*^*floxed/floxed*^*;Dmp1*^*cre*^ mice. In the OTM- group the osteoclast activity in the PDL is slightly increased, but there is no significance compared to the WT group (**f**) (Quantification of TRAP staining, n = 4–5). In addition, the force does not lead to a significant increase in osteoclast activity, as in the control group. Regarding the arrangement of the PDL and the formation of areas of hyalinization there is no difference visible between both groups (**g**) (n = 3). Scale bars = 100 µm. Values are means ± SD. (***P < 0.001).

Having demonstrated the bone phenotype of *Piezo1*^*floxed/floxed*^*;Dmp1*^*cre*^ animals in the absence of an external force, we next asked how orthodontic tooth movement is affected by the loss of *Piezo1* in osteocytes and late osteoblasts. To perform orthodontic tooth movement in *Piezo1*^*floxed/floxed*^*;Dmp1*^*cre*^ mice and control littermates, NiTi-tension springs with a mesial directed force of 0.3N were placed between the upper M1 and the incisors with dental resin for 12 days (Fig. 5a). Subsequently, the extent of tooth movement was determined from the intercoronal distance between the upper M1 and M2 in cross sections of micro computed tomography (µCT) scans (Fig. 5b). It was surprising to find that when the distance was quantified, there were no significant differences between the two groups in terms of the amount of tooth movement (Fig. 5c). To ensure that the tooth movement is not influenced by migration of the second and third molar, we performed additional analysis on these teeth with no significant difference between the groups (Supplementary Fig. 2).

To investigate how the force applied to the molar affected the bone remodeling processes in both groups, we analyzed the number of osteoclasts using TRAP staining in the pressure zones of the mesial and distal root with and without tooth movement (Fig. 5d,e). We counted the osteoclasts covering the bone surface near the root and the osteoclasts directly in the periodontal ligament (PDL). In the pressure zones, tooth movement leads to a significant increase in osteoclast activity in the control group, whereas the number of osteoclasts was not increased in the *Piezo1*^*floxed/floxed*^*;Dmp1*^*cre*^ group (Fig. 5f).

Next, we investigated the extent of hyalinization (a common side effect in dentoalveolar soft tissue as a reaction to heavy forces) using toluidine blue staining, focusing mainly on the area around the distal root (Fig. 5d,e and Supplementary Fig. 3). Hyalinization was evident in both control and *Piezo1*^*floxed/floxed*^*;Dmp1*^*cre*^ animals, and the quantification of the area shows that it does not differ between the two groups (Fig. 5g). Taken together, these results suggest that *Piezo1*-mediated mechanotransduction in osteocytes plays a minor role in orthodontic tooth movement.

## Discussion

Recently, the mechanosensitive ion channels *Piezo1* and *Piezo2* have received much attention in the scientific world, which is also due, among other things, to the awarding of the Nobel Prize in physiology and medicine in the year 2021 to David Julius and Ardem Patapoutian “for their discoveries of receptors for temperature and touch”^12,22^. Both ion channels can be found in a variety of different cell types, but despite nearly identical structures, they seem to have different main functions. For instance, it has been shown that the ion channel *Piezo1* plays an essential role in the mechanosensing of osteoblasts at various stages of differentiation and in the communication between osteoblasts and osteoclasts. *Piezo2* on the other hand, seems to play an important role in mechanosensation when it comes to itch, touch and mechanical pain^23–25^.

In this manuscript, we used an orthodontic tooth movement model in mice lacking *Piezo1* in *Dmp1* expressing cells to investigate whether *Piezo1* plays a role in the mechanosensation of osteocytes during orthodontic tooth movement.

Here we were able to confirm the specific expression of PIEZO1 and PIEZO2 in the dentoalveolar cell types of untreated control animals. PIEZO1 expression was evident in odontoblasts, osteoblasts, and osteocytes. Interestingly, the expression of PIEZO2 was mainly observed in odontoblasts and cementoblasts but not in osteoblasts and osteocytes. The expression pattern of PIEZO1 as well as the low expression of PIEZO2 in bone cells is consistent with previous studies showing that *Piezo2* plays a minor role in mechanically induced bone remodeling in vivo^17,26^.

Interestingly, when we knocked out *Piezo1* in these *Dmp1*-expressing cells, we observed a decrease in bone volume and bone thickness, while the size and shape of the bones did not differ between both groups. Additionally, we also did not observe a difference in osteoblast and osteocyte numbers. Interestingly, mice lacking *Piezo2* in *Dmp1*-expressing cells showed no effect on overall morphology and bone volume (Supplementary Fig. 1). These results are consistent with previously published data investigating the cell-specific deletion of *Piezo1* and *Piezo2* in bone-forming cells to study the bone phenotypes of the mice^17^. The studies indicated that in mice, the elimination of *Piezo1* in cells of the osteoblast lineage led to a reduction in bone volume and impaired bone formation. Cell-specific knockout of *Piezo1* in osteoblast progenitors resulted in severe osteoporotic phenotypes^13,17,18^, whereas cell-specific knockout of *Piezo2* in osteoblast lineage cells did not result in an osteoporotic phenotype^17^. *Piezo2* appears to affect skeletal integrity via expression in proprioceptive neurons and subsequent regulation of skeletal muscle functions rather than having a direct effect in osteoblast lineage cells^27^.

The osteopenic phenotype that occurred in the mice lacking *Piezo1* in bone-forming cells can be linked to reduced bone formation and increased bone resorption due to an increase in the osteoclast differentiation factor RANKL^16^. This is consistent with our finding of increased basal osteoclast activity in the alveolar bone of *Piezo1*^*floxed/floxed*^*;Dmp1*^*cre*^ animals. Another possible mechanism, leading to the decreased bone formation in *Piezo1*^*floxed/floxed*^*;Dmp1*^*cre*^ animals was investigated by Li et al.^13^. Here, *Wnt1* expression (a known bone anabolic ligand^28^) is significantly decreased in *Piezo1*^*floxed/floxed*^*;Dmp1*^*cre*^ mice.

The osteopenic phenotype of mice lacking *Piezo1* in osteoblasts and osteocytes further suggests that they have a reduced ability to respond to mechanical stimulation, which has been confirmed in different anabolic tibia and ulna loading models. In a previous study, it was shown that the osteoanabolic response of *Piezo1*^*floxed/floxed*^*;Dmp1*^*cre*^ animals was reduced, and bone was less responsive to mechanical signals than controls^17^. In our tooth movement model, we see a similar effect, as the loss of *Piezo1* in osteoblasts and osteocytes did not lead to an increase in tooth movement, despite a basal increased osteoclast activity and the subsequent decrease in bone volume. This effect could be explained by a dysfunctional osteoblast-osteoclast interaction, as was described by Wang et al.^16^. However, it is of interest, that the increase in osteoclastogenesis was primary detected in the alveolar bone. We did not observe an increase in osteoclastogenesis in the periodontal bone, which is continuously exposed to masticatory forces. This could indicate that continuous force can reduce the osteoclastogenesis phenotype of Piezo1^floxed/floxed^;Dmp1^cre^ mice. The authors utilized *Piezo1*^*floxed/floxed*^*-Prx1*^*cre*^ mice and control mice in a tail suspension model for their research. Their findings indicated that while the suspension treatment resulted in a rise in osteoclast activity in control animals, it did not surpass the basal increase in the number of osteoclasts in the *Prx1* mice. By that, they underline the importance of *Piezo1* for the modulation of the bone-resorbing activity of osteoclasts^16^. Further evidence for the negligible role of *Piezo1* in osteocytes during OTM could be the areas of hyalinisation, which are the same in both groups. Our initial hypothesis was that a loss of mechanosensing ability in the osteocytes of *Piezo1*^*floxed/floxed*^*;Dmp1*^*cre*^ animals would lead to an altered response and force distribution in the PDL. However, our findings showed that this was not the case; the side effects of tooth movement were just as pronounced in *Piezo1*^*floxed/floxed*^*;Dmp1*^*cre*^ mice as in control animals.

Our results suggest that mechanically induced bone remodeling using orthodontic forces is not dependent on *Piezo1* expression in hard tissue. However, since we performed our tooth movement model for a period of 12 days, it is reasonable to assume that a more extended period could result in a significant difference in bone remodeling since long-term effects of *Piezo1* expression on bone remodeling may not be fully captured. Further research with more extended observation periods is needed to fully understand the role of *Piezo1* in bone remodeling in hard tissue.

Furthermore, it is important to note that the mechanosensitivity of osteocytes is not solely dependent on mechanosensing ion channels, but also on other structures such as primary cilia, integrins, and G-proteins. These structures play a crucial role in enabling the osteocytes to respond to mechanical forces and thus could influence tooth movement^29^. Furthermore, targeting osteoblast precursor using a cell-specific knockout of *Piezo1* in *Runx2* expressing cells might also lead to a more pronounced impact on tooth mobility. However, we did not use this mouse line since a previous study showed that *Piezo1*^*floxed/floxed*^*;Runx2*^*cre*^ mice were prone to rib fractures starting from day P5 due to their severe osteoporotic phenotype^17^.

Additionally, the involvement of mechanosensing ion channels within the periodontal soft tissue and its role during tooth movement might be more critical than previously anticipated^30–33^. Here, the role of *Piezo1* in fibroblasts of the periodontal ligament has been investigated in previous studies. Jin et al. stated in their in vitro study that pressure on PDL fibroblasts leads to an increased expression of *Piezo1*^34^. The same effect was later confirmed in vivo using a tooth movement model in control mice^35,36^. In addition, Jiang et al. confirmed an increase of *Piezo1* expression in the tension zone of the PDL as a reaction to mechanical force and furthermore showed that *Piezo1* was important for the expression of osteogenic markers^20^. As part of the research, the investigators employed the mechanosensitive ion channel inhibitor GsMTx4 to explore its impact on tooth movement^37^. In vitro, periodontal cells treated with GsMTx4 and subsequently exposed to a static pressure showed a reduction in RANKL expression and a concomitant decrease in osteoclastogenesis^34–36^. In vivo a systemical treatment with GsMTx4 led to reduced tooth movement in rats^20^. However, in this particular setup, all cells of the dentoalveolar apparatus are affected when the inhibitor is administered systemically. Therefore, it is impossible to predict the importance of individual cell types in the overall process.

Overall, our results suggest that despite *Piezo1* being crucial for osteoclast function, it may be dispensable for mechanical sensing of bone remodeling.

## Materials and methods

### Mice

Generation and genotyping *Piezo1*^*floxed/floxed*^ and *Piezo2*^*floxed/floxed*^ mice have been described previously^38,39^. For the study, a mouse line with a 14 kb *Dmp1* promoter fragment (JAX #023047) was used to drive specific expression of *Cre* Recombinase. The generation of the mouse line has been previously described by Lu et al.^40^. To rule out any possible influence of genetic background all analyses were performed with the corresponding *Cre*-negative littermate controls. These Cre-negative littermates are named control animals in the manuscript.

For this particular study, we utilized a total of 12 mice to that exhibited orthodontic tooth movement, including 6 control mice and 6 *Piezo1*^*floxed/floxed*^*;Dmp1*^*cre*^ mice. In addition to evaluating tooth movement, we also analyzed the skull phenotype of 3 *Piezo2*^*floxed/floxed*^*;Dmp1*^*cre*^ mice.

Generation of *Dspp*-^Cerulean^;*Dmp1*^*Cherr*y^ reporter mice has been described by Vijaykumar et al.^41^ and experimental animals were kindly provided by Jan Krivanek (Department of Histology and Embryology, Faculty of Medicine, Masaryk University, Brno, Czech Republic).

Mice were housed in a specific pathogen-free environment with a 12-h light/dark cycle, 45% to 65% relative humidity, and 20 °C to 24 °C ambient temperature in open or individually ventilated cages with wood shavings bedding and nesting material in groups not surpassing six animals. Mice had ad libitum access to tap water and standard rodent chow. Animal care and handling as well as all experimental procedures were performed in accordance with all national and european guidelines. All methods were performed in accordance with the relevant guidelines and regulations. The study is reported in accordance with ARRIVE guidelines.

The animal procedures were approved by the local animal care committees of the University Medical Center Hamburg-Eppendorf and the University of Leipzig Medical Center (TVA 062/20 and TVV 03/21).

### Orthodontic appliance

The experiment was performed as a split-mouth model, with the untreated jaw side as an internal control. The experimental procedure was described before^21^. Mice were anesthetized with a solution of ketamine (40 mg kg^−1^ bw Ketamin-S), xylazine 2% (16 mg kg^−1^ bw), and 0.9% NaCl. After etching the left first maxillary molar and the incisors (HS-etch gel 37%, Henry Schein Dental, Langen, Germany), bonding was applied to the teeth, and the distal end of the nitinol open coil spring (Sentalloy Open Coil Spring, Dentsply Sirona, Pennsylvania, USA) was bonded to the surface of the left first maxillary molar with a force of 30 centinewtons using light-cured resin (Estelite Flow Quick, Tokuyama Dental Corp., Tokyo, Japan). After orthodontic treatment, the animals were monitored on a heating mat until their emergence. Animals were euthanized by CO2 inhalation after 12 days of OTM. The heads were fixed in 4% PFA at 4 °C for 24 h and then transferred to 80% ethanol.

### Micro-computed tomography

The skulls were scanned with a voxel resolution of 15 µm/5 µm using micro-computed tomography (µCT 40, Scanco Medical, Brüttisellen, Switzerland/Skyscan 1172, Bruker, Kontich, Belgium) as described previously^42,43^. Wall Thickness Analysis and Bone Volume measurements were performed on 3-dimensional reconstructions of the scans with the software Avizo (FEI, USA). Horizontal Alveolar bone loss (CEJ-ABC) was measured on 2-dimensional microCT-pictures with ImageJ as previously described^44^.

### Histology

After performing µCT-analysis, the upper jaws were prepared for histological examination. They were decalcified using 10% EDTA for 14 days, while the reagent was changed once a week. Afterward, the upper jaws were cut in the parasagittal plane. The halves were dehydrated in ascending alcohol concentrations and embedded in paraffin. Sections of 5 µm thickness were prepared using a microtome. Toluidine Blue staining was performed as previously described, and TRAP staining was performed using the Sigma Aldrich TRAP Kit (387A, Sigma Aldrich, Missouri, USA) according to the manufacturer's instructions.

All histomorphometric analyses were performed with QuPath 0.4.2.^45^ and ImageJ 1.52 (National Institutes of Health, Betheseda, MD, USA) and its implication BoneJ 1.4.3^46^. Trap Staining was quantified as the Number of Osteoclasts per bone area in the pressure zone of the mesial and distal root and as well in the interrradicular bone with QuPath.

### Fluorescence immunohistology

Immunohistology was carried out on paraffin sections. For this, the sections were deparaffined in xylene baths. The samples were rehydrated in descending alcohol series for 2 min each, followed by a short wash in distilled water. Afterward, antigen retrieval was performed with Proteinase K (0,1ul/ml in Tris–HCl) for 20 min. After rinsing in PBS the staining was performed with PIEZO1 (MA5-32876 1:50 in Dako Solution (# s1699)) for 24 h at RT and PIEZO2 (PA5-72975 1:100 in Dako Solution (# s1699)) for 24 h at RT. After rinsing in PBST (0,1% Tween) the secondary antibodies M555 (Invitrogen A28180) and Rb647 (Invitrogen A32733) were diluted in Dako solution 1:1000 and put on the slides for 1 h at RT. DAPI staining (1:1000 in PBST) was perfomed for 5 min at RT. After rinsing, the slides were mounted in Mowiol 4–88 (Kuraray Europe GmbH, Germany) Mounting Medium. Sections were examined and imaged using the Leica Thunder Imager 3D Assay (Leica Microsystems, Wetzlar, Germany) with filter cubes optimized for the detection of GFP (HQ470/40ex, HQ525/50em, HQ495lp beam splitter), TXR (HQ560/40ex, HQ630/75em, HQ585lp beam splitter), Y5 (HQ620/60ex, HQ700/75em, HQ660lp beam splitter) and DAPI (HQ405/60ex, HQ470/40em, HQ455lp beam splitter).

### Statistical analysis

The statistical analysis was performed with GraphPad PRISM9 (GraphPad Software, San Diego, USA). Results were expressed as the mean ± SD. Two-sided *t*-test and one-way ANOVA with Bonferroni post hoc test were used for multiple group comparisons. *P* values below 0.05 were considered statistically significant.

## Supplementary Information

Supplementary Figures.

## Data Availability

Access to data will be provided to researchers subject to submission of a research proposal and signing a Data Use Agreement. Interested researchers can request access to the data by contacting the corresponding author.
