# Supplementary material for: Mechanical-induced bone remodeling does not depend on Piezo1 in dentoalveolar hard tissue
Source: Sci Rep. 2023 Jun 12;13:9563. doi: 10.1038/s41598-023-36699-9 (PMC10261143; doi:10.1038/s41598-023-36699-9)
Supplement: Supplementary file 1 — Supplementary Figures. [file 41598_2023_36699_MOESM1_ESM.pdf]

Supplementary Figures

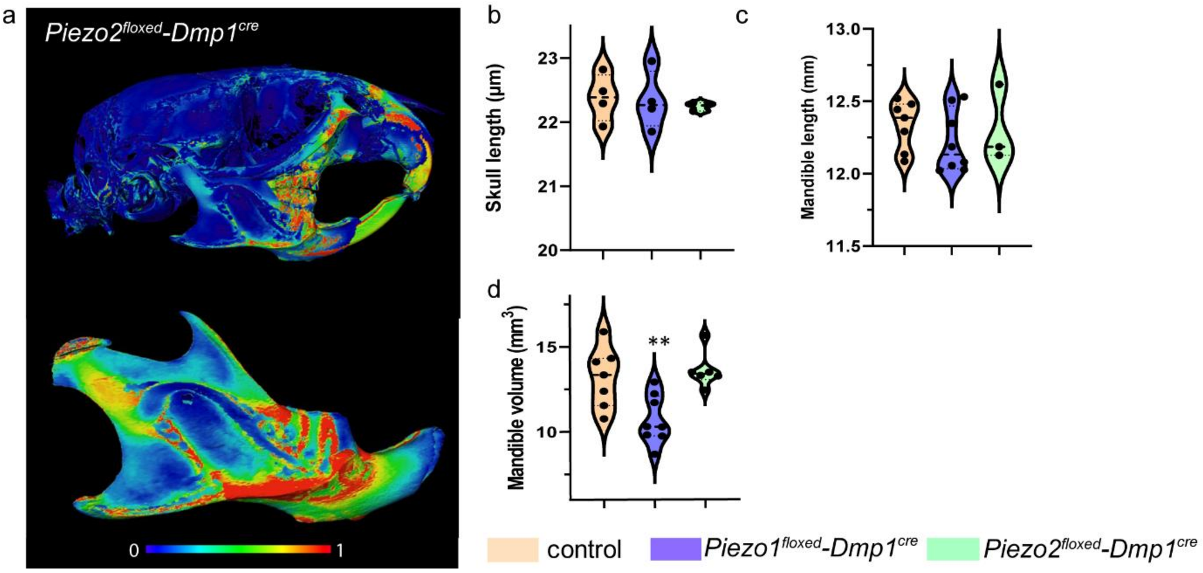

Suppl. Figure 1: Deletion of *Piezo2* in *Dmp1* expressing cells does not affect the bone phenotype of the skull.

3-dimensional reconstruction of Micro-Computed Tomography scans of *Piezo2*$^{floxed/floxed}$*;Dmp1*^{cre} mice (**a**). The wall thickness analysis of the skull and the mandible does not show differences compared to control animals (comp. with Fig **3a and b**.) The skull and mandible length does not differ between the groups (**b and c**). The mandible volume of *Piezo2*$^{floxed/floxed}$*;Dmp1*^{cre} mice is not altered compared to their control littermates whereas the bone volume of the mandible is significantly decreased (**d**). Values are means ± SD.*(**P <0.01)*

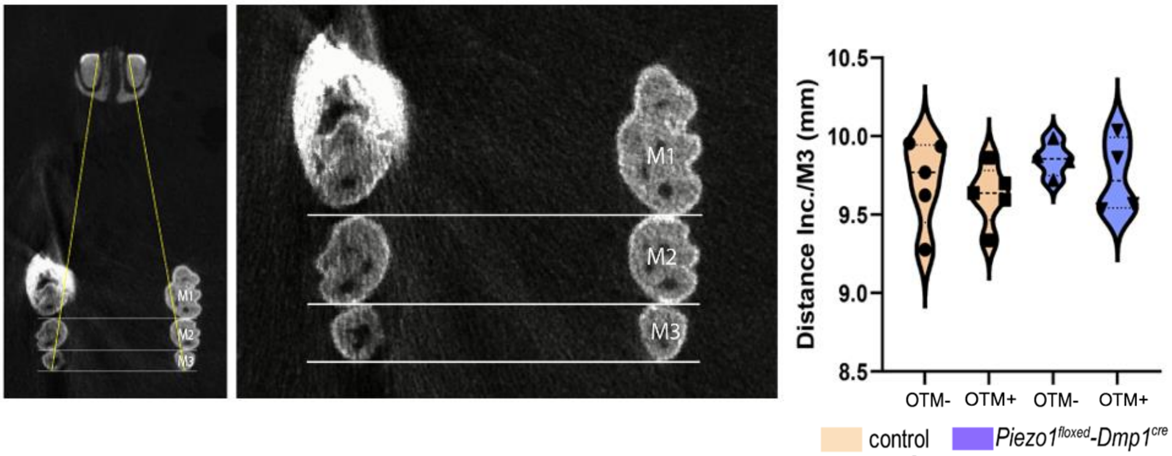

Suppl. Figure 2: Mesialization of the M1 does not lead to a migration of the M2 and M3. The figure shows a horizontal section through the skull of a mouse that has undergone tooth movement. On the left side you can see that M1 has been mesialised. Compared to the control site where a strong contact between the crowns is visible, the contact point between M1 and M2 is dissolved in the OTM+ site. The horizontal white lines between M1-M3 are perpendicular to the sagittal plane, to underline that no migration of the molars is evident. The graph on the right also shows the quantification of the distance between the incisors and the most distal point of M3, which is also shown by the yellow line.

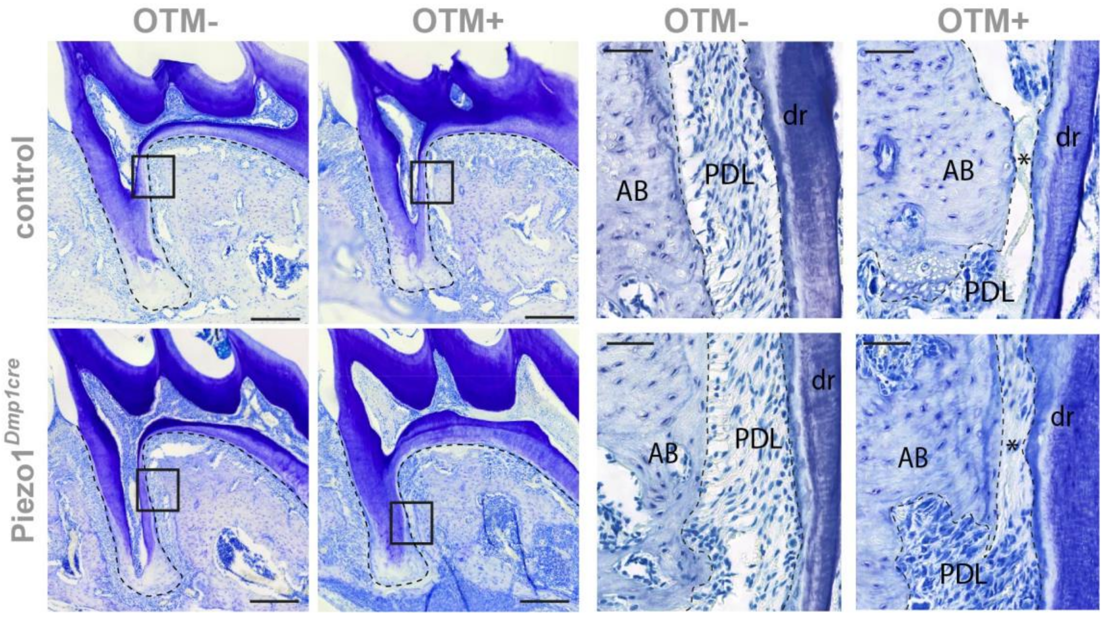

Suppl. Figure 3: Regions of interest for quantification of hyalinization areas in the pressure zones of the PDL. The figure shows areas of hyalinization in control and *Piezo1*$^{floxed/floxed}$*;Dmp1*^{cre} mice that occur after 12 days of tooth movement. Most of the hyalinizations occur in the pressure zone of the distal root (black boxes), as the pressure in these areas is relatively high. Scale bars=200µm. The zoom ins on the right side show higher magnification of these areas. Scale bars=100µm. The amount of the areas of hyalinization does not differ between both groups.
